# Identification of Gαi3 as a promising molecular oncotarget of pancreatic cancer

**DOI:** 10.1038/s41419-024-07079-6

**Published:** 2024-09-30

**Authors:** Jian-zhuo Jiang, Yin-biao Qiao, Xiao-ren Zhu, Qian-hui Gu, Jing-jing Lu, Zhen-yu Ye, Lu Xu, Yuan-yuan Liu

**Affiliations:** 1 Clinical Research and Lab Center, Affiliated Kunshan Hospital of Jiangsu University, Kunshan, China; 2 General Surgery, Cancer Center, Department of Colorectal Surgery, Zhejiang Provincial People’s Hospital, Hangzhou, China; 3 Department of Radiotherapy and Oncology, Affiliated Kunshan Hospital of Jiangsu University, Kunshan, China; 4 Department of General Surgery, The Second Affiliated Hospital of Soochow University, Suzhou, China; 5 Department of general surgery, The first affiliated hospital of Soochow university, Suzhou, China

**Keywords:** Mechanisms of disease, Targeted therapies

## Abstract

The increasing mortality rate of pancreatic cancer globally necessitates the urgent identification for novel therapeutic targets. This study investigated the expression, functions, and mechanistic insight of G protein inhibitory subunit 3 (Gαi3) in pancreatic cancer. Bioinformatics analyses reveal that Gαi3 is overexpressed in human pancreatic cancer, correlating with poor prognosis, higher tumor grade, and advanced classification. Elevated Gαi3 levels are also confirmed in human pancreatic cancer tissues and primary/immortalized cancer cells. Gαi3 shRNA or knockout (KO) significantly reduced cell viability, proliferation, cell cycle progression, and mobility in primary/immortalized pancreatic cancer cells. Conversely, Gαi3 overexpression enhanced pancreatic cancer cell growth. RNA-sequencing and bioinformatics analyses of Gαi3-depleted cells indicated Gαi3’s role in modulating the Akt-mTOR and PKA-Hippo-YAP pathways. Akt-S6 phosphorylation was decreased in Gαi3-depleted cells, but was increased with Gαi3 overexpression. Additionally, Gαi3 depletion elevated PKA activity and activated the Hippo pathway kinase LATS1/2, leading to YAP/TAZ inactivation, while Gαi3 overexpression exerted the opposite effects. There is an increased binding between Gαi3 promoter and the transcription factor TCF7L2 in pancreatic cancer tissues and cells. Gαi3 expression was significantly decreased following TCF7L2 silencing, but increased with TCF7L2 overexpression. In vivo, intratumoral injection of Gαi3 shRNA-expressing adeno-associated virus significantly inhibited subcutaneous pancreatic cancer xenografts growth in nude mice. A significant growth reduction was also observed in xenografts from Gαi3 knockout pancreatic cancer cells. Akt-mTOR inactivation and increased PKA activity coupled with YAP/TAZ inactivation were also detected in xenograft tumors upon Gαi3 depletion. Furthermore, bioinformatic analysis and multiplex immunohistochemistry (mIHC) staining on pancreatic cancer tissue microarrays showed a reduced proportion of M1-type macrophages and an increase in PD-L1 positive cells in Gαi3-high pancreatic cancer tissues. Collectively, these findings highlight Gαi3’s critical role in promoting pancreatic cancer cell growth, potentially through the modulation of the Akt-mTOR and PKA-Hippo-YAP pathways and its influence on the immune landscape.

## Introduction

Pancreatic cancer persists as a predominant cause of cancer-related mortality worldwide, with its incidence doubling over the past two decades [1]. Characterized by advanced presentation, including systemic metastasis and inoperability at diagnosis, pancreatic cancer is associated with a notably poor prognosis, evidenced by overall five-year survival less than 10% [2–4]. The efficacy of conventional treatment modalities, such as radiotherapy and gemcitabine-based chemotherapy, remains constrained for patients with unresectable pancreatic cancers [5, 6]. Despite advancements in genomic diagnostics and introduction of molecularly targeted therapies improving outcomes for advanced pancreatic cancer patients, the overall survival remains grim [7].

The pathogenesis of pancreatic cancer is significantly influenced by the aberrant expression and persistent activation of various cell surface receptor tyrosine kinases (RTKs), including EGFR (epidermal growth factor receptor), VEGFR (vascular endothelial growth factor receptor), and PDGFR (platelet-derived growth factor receptor), and their downstream oncogenic pathways, contributing to disease initiation, progression and chemoresistance [8–10]. Co-activation of multiple RTKs leads to the continuous activation of oncogenic signaling pathways, facilitating unrestrained growth of cancer cells [8, 11]. Although combination therapies of tyrosine kinase inhibitors (TKIs) with gemcitabine are utilized as initial treatment strategies for pancreatic cancer, the efficacy of TKIs is hampered by the eventual development of drug resistance [8, 12]. Thus, there is a critical need to identify novel molecular targets essential for pancreatic cancer progression and to develop therapeutic strategies that can target multiple RTKs simultaneously.

The inhibitory heterotrimeric guanine nucleotide-binding proteins, Gαi proteins, comprising three subunits, Gαi1, Gαi2 and Gαi3 [13, 14], traditionally associate with G protein-coupled receptors (GPCRs) to inhibit adenylate cyclase (AC) activation, thereby reducing cellular cyclic AMP (cAMP) levels [13]. Notably, our research group has demonstrated that Gαi1 and Gαi3 are pivotal in mediating signals from various RTKs, including EGFR (epidermal growth factor receptor), FGFR (fibroblast growth factor receptor), KGFR (keratinocyte growth factor receptor), c-Kit (stem cell factor receptor), TrkB (the BDNF receptor), and VEGFR2 (vascular endothelial growth factor receptor 2) [15–20], acting as key signaling proteins in transducing oncogenic signaling cascades (such as Akt-mTORC1 and Erk-MAPK). Moreover, Gαi1/3 can also associate with several other non-RTK receptors, including RSPO3 (R-spondin 3)’s receptor LGR4 (leucine-rich repeat-containing G-protein-coupled receptor 4) [21], Netrin-1’s receptor CD146 [22] and IL-4’s receptor [23], transducing downstream Akt-mTOR signaling activation. This indicates the potential of Gαi proteins as significant oncogenic genes and therapeutic targets in cancer [15–19]. Recent studies, including our own, have identified overexpression of Gαi proteins in various human malignancies, underscoring their critical roles in tumorigenesis and cancer progression [16, 24–27]. This study aims to explore the expression and functional roles of Gαi3 in pancreatic cancer.

## Materials and methods

### Reagents and antibodies

Primary antibodies were utilized as follows: anti-LATS1/2 (1:1000; #DF7517, Affinity), anti-p-LATS1/2 (1:1000; #AF8163, Affinity), anti-PKA C-α (1:1000; #4782, Cell Signaling Technology), anti-p-PKA C Thr-197 (1:1000; #4781, Cell Signaling Technology), anti-YAP1 (1:1000; #205270; Abcam), anti-p-YAP Ser-127 (1:2000; #13008, Cell Signaling Technology), anti-p-TAZ (1:2000; #AF4315, Affinity).Other antibodies were described previously [16, 20, 25, 28]. H-89 was from MCE (Shanghai, China), other reagents were provided by Thermo-Fisher Invitrogen (Suzhou, China).

### Cells, human tissues, and tissue microarray

The immortalized pancreatic cancer cell lines, BxPC-3 and CFPAC-1, were acquired from the Institute of Biochemistry and Cell Biology, Chinese Academy of Sciences (Shanghai, China). These cell lines were maintained in Dulbecco’s Modified Eagle Medium (DMEM) enriched with 10% fetal bovine serum (FBS) and 1% penicillin-streptomycin (Pen/Strep). The methodology for the isolation and culture of primary human pancreatic cancer cells (“priPC-1”,“priPC-2”and “priPC-3”) alongside the primary human pancreatic epithelial cells (“pEpi”) has been elaborated in prior studies [29–31]. A tissue microarray specific to human pancreatic cancer was procured and analyzed by Shanghai Outdo Biotech Co. Ltd. (Shanghai, China). Fresh pancreatic cancer tissues and adjacent normal pancreatic tissues were collected from twelve primary pancreatic cancer patients at the Affiliated Kunshan Hospital of Jiangsu University and the Second Affiliated Hospital of Soochow University. Written informed consent was obtained from each patient. The experimental protocols employed in this study received approval from the Ethics Review Board of The Affiliated Kunshan Hospital of Jiangsu University, adhering to the ethical guidelines of the Helsinki Declaration.

### Genetic alteration of Gαi3 or TCF7L2

The silencing of Gαi3 via short hairpin RNA (shRNA), the knockout (KO) of Gαi3 utilizing the CRISPR/Cas9 gene editing technique, and the forced overexpression of Gαi3 facilitated by a lentiviral construct were detailed in our prior publications [16, 22, 25, 28]. The Gαi3-shRNA-expressing adeno-associated virus (aav) for in vivo studies was reported in our previous studies [25, 28]. The TCF7L2 shRNA-sequence (targeting 5’-*CCTCCGCACCCTCCAGATATATC*-3’) and the TCF7L2-expressing cDNA sequence ([NM_030756.5]) were custom-synthesized by Genechem in Shanghai, China. These sequences were separately integrated into the GV369 vector [26, 32, 33]. This vector, together with the lentivirus packaging vectors, was co-transfected into HEK-293 cells, facilitating the production of lentivirus. The resulting lentivirus was then filtered, concentrated at an MOI of 10.0, and introduced to cultured pancreatic cancer cells. Following treatment with puromycin to select for stable cells, the expression of TCF7L2 in these cells was consistently verified.

### Constitutively-active mutant Akt1 (caAkt1)

Primary human pancreatic cancer cells were infected with the lentivirus bearing the constitutively active S473D mutant of Akt1 (“caAkt1”), as well as an empty vector-expressing virus, as described previously [21, 32, 33]. Stable cells expressing caAkt1 were established through selection using puromycin.

### Multicolor immunohistochemistry

The pancreatic cancer tissue microarray, provided by Shanghai Outdo Biotech Co., Ltd. (Shanghai, China), underwent multicolor immunohistochemistry (mIHC) staining using the AlphaTSA 7 color fluorescent staining kit (AlphaX, Beijing, China), adhering to the manufacturer’s instructions. Tyramide signal amplification (TSA) immunofluorescence (IF) technique facilitated the detection of target antigen expression within each sample. Antigens within the tissue cells were identified using specifically labeled antibodies, with the resultant fluorescence and coloration highlighting the in situ antigen distribution. The tissue microarray was heated at 60 °C for 30 min and subsequently dehydrated using an AlphaPainter X30 Multi-target pathology stain machine (AlphaX Bio, Beijing, China), preparing it for antigen retrieval. Endogenous peroxidase activity was quenched by applying a peroxidase-blocking solution for 15 minutes.

For IF staining, the microarray was incubated with primary antibodies against Gαi3 (1:200, ab154024; Abcam), CD163 (1:200, ZM0428, ZSGB-BIO), CD68 (1:400, ZM0060, ZSGB-BIO), PD-L1 (1:100, CST13684, Cell Signaling Technology), HLA-DR (1:2000, ab92511; Abcam), and PANCK (1:100, ZM0069, ZSGB-BIO) at room temperature for one hour. After washing, the microarray was treated with secondary antibodies for 15 min. A TSA dye working solution was then applied and allowed to incubate for 10 min at room temperature. Images were taken under the scanning system Axioscan 7 (ZEISS, Oberkohen, Germany), quantitative analysis and data statistical analysis were performed for each target eventually.

### Immunofluorescence

Cells were seeded in laser confocal dishes until achieving ~50% confluence. Subsequently, cells were washed with PBS for three times and then fixed with 4% paraformaldehyde for 30 min. Permeabilization followed by using 0.5% Triton-X for 10 min. Bovine Serum Albumin (BSA, Solarbio, Beijing, China) was employed to block non-specific binding sites for 1 h at room temperature. Cells were then incubated with primary antibodies at 4 °C overnight. Followed by incubation with a fluorescently-labeled secondary antibody. Nuclei staining was carried out using DAPI for 5 min, and the samples were subsequently sealed and visualized under a confocal microscope (ZEISS, Oberkohen, Germany)

### Cellular functional studies and gene/protein expression detections

Western blotting, quantitative real-time PCR (qPCR), cell viability assays using Cell Counting Kit-8 (CCK-8), EdU (5-ethynyl-20-deoxyuridine) incorporation for cell proliferation analysis and colony formation assay, as well as “Transwell” in vitro migration and “Matrigel Transwell” in vitro invasion assays have been comprehensively delineated in our previous studies [16, 30, 31]. The protocols for siRNA experiments (100 nM, transfected for 48 h) are outlined in detail in our previous study [26]. The verified siRNAs used were purchased from Genechem. Figure S1 listed all uncropped blotting images.

### Cytoplasmic/nuclear protein extraction

Protein extraction was performed using the “Nuclear and Cytoplasmic Protein Extraction Kit” (Beyotime, Shanghai, China), following the manufacturer’s instructions. Cells were first washed with PBS and collected via centrifugation. For cytoplasmic protein isolation, 200 µL of Cytoplasmic Protein Extraction Reagent A (with 1 mM PMSF) was added to the cell pellets, mixed, incubated on ice for 15 min for lysis, followed by the addition of 10 µL of Reagent B and another 1-min incubation. Centrifugation at 16,000×*g* for 5 min at 4 °C separated the cytoplasmic proteins. Nuclear proteins were extracted by adding 50 µL of Nuclear Protein Extraction Reagent (1 mM PMSF) to the remaining pellets, mixing over 30 min, and centrifuging at 16,000×*g* for 10 min at 4 °C. Protein concentrations for both extracts were determined using the BCA Assay kit (Thermo-Fisher Invitrogen, Suzhou, China)

### Chromatin immunoprecipitation

The methodology for the Chromatin immunoprecipitation (ChIP) assay was based on previously established protocols [26, 34]. Briefly, lysates from either cells or tissues were homogenized, followed by the fragmentation of genomic DNA [27]. These lysates were then subjected to dilution and immunoprecipitation using an anti-TCF7L2 (Transcription Factor 7-Like 2) antibody, enabling the isolation of TCF7L2-bound DNA fragments. Subsequently, the association between TCF7L2 and the proposed Gαi3 promoter DNA (*AGATCAAAG*) sequence was assessed through quantitative PCR (qPCR), with results normalized to control values.

### Xenograft studies

The animal experiments received the necessary approvals from the IACUC (Institutional Animal Care and Use Committee) and the Ethics Review Board of The Affiliated Kunshan Hospital of Jiangsu University. Female BALB/c nude mice (body weight 16 ± 1.5 g) aged 5–6 weeks were acquired from the Animal Center of Jiangsu University and were housed under standardized indoor conditions. Primary human pancreatic cancer cells were prepared in a suspension (five million cells per mouse) in a DMEM/Matrigel mixture and were subcutaneously inoculated into the right flank of each mouse. Three weeks post-injection, the xenografts reached an approximate volume of 100 mm³. The mice were subsequently divided into two distinct groups, with each group comprising six mice (*n* = 6). The mice thereafter received intratumoral injections of either an adeno-associated virus (aav) bearing shRNA against Gαi3 (“aav-shGαi3-s1” [25, 28]) or a comparative amount of a scramble control shRNA vector (“aav-shC”). Tumor volume measurements were conducted using the formula (length × width²)/2. Additionally, mice body weights were monitored, and daily tumor growth rates were calculated following the described methodologies [25, 28]. In a parallel study, nude mice were subcutaneously injected with either ko-Gαi3 priPC-1 cells or control Cas9-C priPC-1 cells (twenty million cells per mouse), with priPC-1 xenograft sizes being evaluated after 30 days. The immunohistochemistry (IHC) analysis of the xenograft tissues was conducted in accordance with methodologies outlined in prior studies [24, 33].

### Statistical analysis

In vitro experiments were repeated with a minimum of five biological replicates. The results were presented as mean ± standard deviation (SD). For statistical evaluations, GraphPad Prism version 9.5 software was utilized. Comparative analysis between two distinct groups employed the two-tailed Student’s t-test. For comparisons involving more than two groups, a one-way analysis of variance (ANOVA) followed by Tukey’s post hoc test was applied. A *P*-value of less than 0.05 was deemed indicative of statistical significance.

## Results

### The bioinformatic analysis supports Gαi3 overexpression in pancreatic cancer

**Fig. 1:**
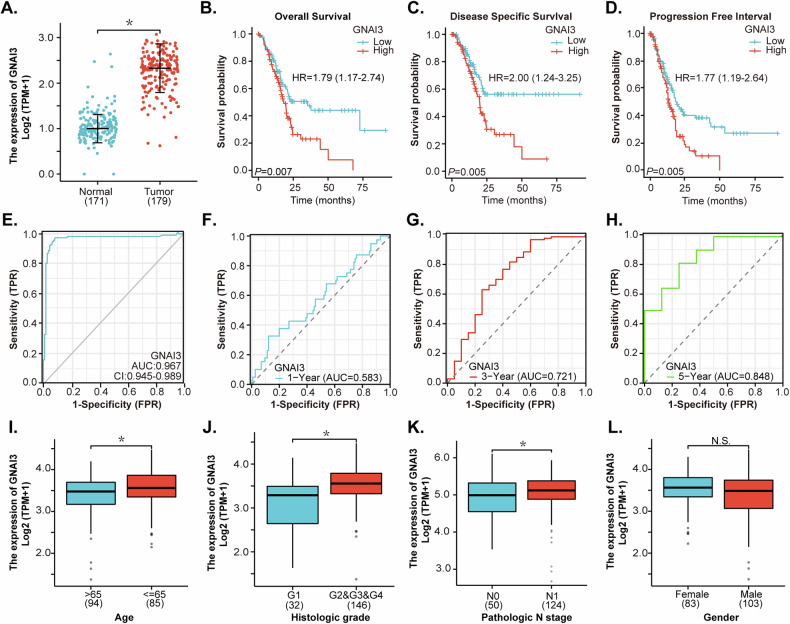
The bioinformatic analysis supports Gαi3 overexpression in pancreatic cancer. The Cancer Genome Atlas (TCGA) PAAD database shows the mRNA levels of *Gαi3* (*GNAI3*) in pancreatic cancer tissues (“Tumor”, *n* = 171) and normal pancreatic tissues (“Normal”, *n* = 179) (**A**), and the Kaplan–Meier Survival analyses of overall survival (**B**), disease-specific survival (**C**), and progression-free survival (**D**) were performed based on *Gαi3* expression in TCGA-PAAD dataset. The receiver operating characteristic(ROC) curves for evaluating the prognostic potential of *Gαi3* overexpression in relation to the overall survival probability of pancreatic cancer patients were shown (**E**–**H**). Subgroup analyses explore the correlation between *Gαi3* expression levels and distinct clinical characteristics of pancreatic cancer patients (**I**–**L**). “TPM” refers to transcripts per million, “AUC” denotes the area under the curve, “HR” represents the hazard rate, “TPR” is the true positive rate, and “FPR” stands for the false positive rate. *P < 0.05. “N.S.” stands for non-statistical difference (*P* > 0.05).

The bioinformatic analysis was performed to evaluate the expression levels of *Gαi3* (*GNAI3*) in human pancreatic cancer utilizing The Cancer Genome Atlas (TCGA) database. This analysis encompassed a total of 179 pancreatic cancer tissues (“Tumor”) and 171 normal pancreas tissues (“Normal”) (Fig. 1A). As shown, *Gαi3* transcripts are significantly elevated in pancreatic cancer tissues compared to those in normal pancreatic tissues (Fig. 1A). Further investigation indicated that overexpression of *Gαi3* is associated with decreased overall survival (hazard ratio [HR]: 1.79, *P* = 0.007) (Fig. 1B), disease-specific survival (DSS; HR: 2.00, *P* = 0.005) (Fig. 1C), and progression-free survival (PFS; HR: 1.77, *P* = 0.005) (Fig. 1D). The Receiver Operating Characteristic (ROC) curve is a graphical tool utilized in cancer diagnosis to assess the performance of potential diagnostic marker [35]. It plots the true positive rate (TPR) against the false positive rate (FPR) at threshold settings, providing insight into the test’s ability to distinguish between individuals with cancer and those without it. ROC curve analysis suggested that *Gαi3* overexpression might serve as a predictive marker for pancreatic cancer diagnosis, achieving an area under the curve (AUC) of 0.967 (Fig. 1E). Furthermore, AUC values for predicting 1-year, 3-year, and 5-year survival are 0.583, 0.721, and 0.848, respectively (Fig. 1F–H). Clinical subgroup analysis revealed a significant correlation between *Gαi3* overexpression in pancreatic cancer and patients’ age (Fig. 1I), histological grade (Fig. 1J), and N classification (Fig. 1K), whereas no correlation was found with patients’ gender (Fig. 1L). The bioinformatics findings indicate that *Gαi3* is upregulated in pancreatic cancer, showing a correlation with poorer overall survival and other significant clinical parameters among patients.

### Gαi3 is overexpressed in pancreatic cancer tissues and different pancreatic cancer cells

**Fig. 2:**
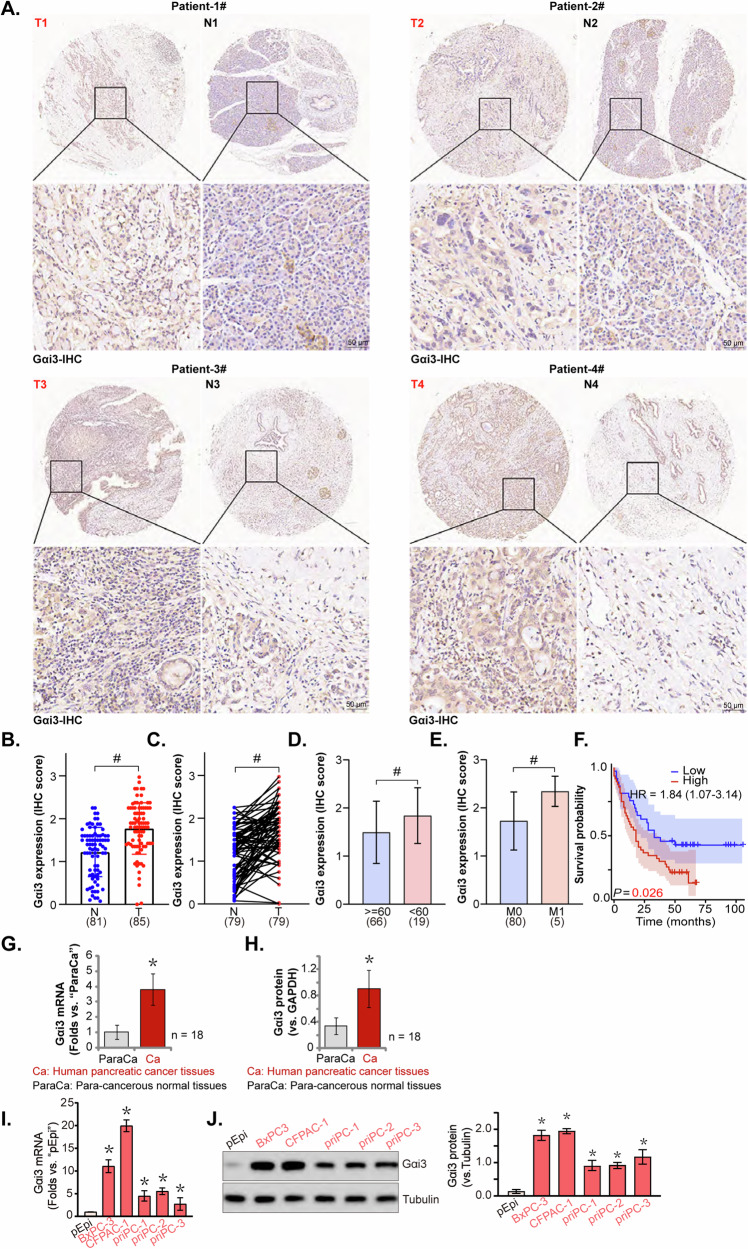
Gαi3 is overexpressed in pancreatic cancer tissues and different pancreatic cancer cells. Immunohistochemistry (IHC) staining was performed on a tissue microarray from pancreatic cancer patients. Representative Gαi3 IHC images from four pancreatic cancer patients (Patient-1#/-2#/-3#/-4#) were displayed (**A**). A comprehensive evaluation of Gαi3 expression was conducted using IHC scores from the tissue microarray. Gαi3 expression levels in unpaired samples, comprising pancreatic cancer tissues (“T”, *n* = 85) and normal tissues (“N”, *n* = 81), and paired samples, comprising pancreatic cancer tissues (“T”, *n* = 79) and their corresponding normal tissues (“N”, *n* = 79, were shown (**B**, **C**). Subgroup analyses showed the Gαi3 expression in different clinical categories of pancreatic cancer patients (**D**, **E**). Kaplan Meier Survival analyses based on Gαi3 expression in pancreatic cancer patients were shown (**F**). *Gαi3* mRNA (**G**) and protein (**H**) expression in the surgically-resected fresh pancreatic cancer tissues (“Ca”) and paired adjacent normal tissue (“ParaCa”) of a total of 18 different patients (*n* = 18) was tested, with results quantified. *Gαi3* mRNA (**I**) and protein (**J**) expression in the described pancreatic cancer cells and primary human pancreatic epithelial cells (“pEpi”) was shown, with results quantified. The data were presented as mean ± standard deviation (SD). *P < 0.05 vs.“N” tissues/“ParaCa”/“pEpi”. ^#^*P* < 0.05. Scale bar = 50 μm.

The expression of Gαi3 in human pancreatic cancer tissues was investigated using a tissue microarray comprising 85 pancreatic cancer tissues (“T”) and 81 normal pancreas tissues (“N”). Immunohistochemistry (IHC) staining was employed to visualize Gαi3 protein expression levels. Representative IHC images from four patients (labeled as Patient-1#/-2#/-3#/-4#) demonstrated elevated Gαi3 expression in pancreatic cancer tissues compared to normal pancreatic tissues (Fig. 2A). Further analysis, both on unpaired (85 pancreatic cancer tissues versus 81 normal tissues) and paired samples (79 pancreatic cancer tissues matched with 79 normal tissues), consistently indicated significant upregulation of Gαi3 protein in pancreatic cancer tissues (Fig. 2B, C). Additionally, a correlation was observed between Gαi3 overexpression and patients’ age (Fig. 2D), tumor metastasis status (Fig. 2E), and poorer overall survival (Fig. 2F). These results further corroborated the bioinformatics results.

We obtained freshly pancreatic cancer tissues (“Ca”) from patients undergoing surgery at local hospitals. These cancer tissues were accompanied by adjacent normal tissue (“ParaCa”). Results from qRT-PCR (Fig. 2G) and Western Blotting (Fig. 2H) confirmed significantly higher levels of *Gαi3* mRNA and protein expression in pancreatic cancer tissues compared to the adjacent normal tissues. Subsequent investigations focused on Gαi3 expression in various human pancreatic cancer cells, including three patient-derived primary pancreatic cancer cell lines (“priPC-1”, “priPC-2” and “priPC-3” [29]) and the immortalized BxPC-3 and CFPAC-1 cell lines. Quantitative analysis revealed that both primary and immortalized pancreatic cancer cells exhibit significantly higher levels of *Gαi3* mRNA compared to normal primary human pancreatic epithelial cells (“pEpi” [29]) (Fig. 2I). Similarly, Gαi3 protein levels were found to be elevated in these primary and established pancreatic cancer cells (Fig. 2J). Collectively, these results clearly confirmed that Gαi3 is overexpressed in human pancreatic cancer tissues and different pancreatic cancer cells.

### Gαi3 silencing by shRNA inhibits pancreatic cancer cell growth, cell cycle progression, and mobility

**Fig. 3:**
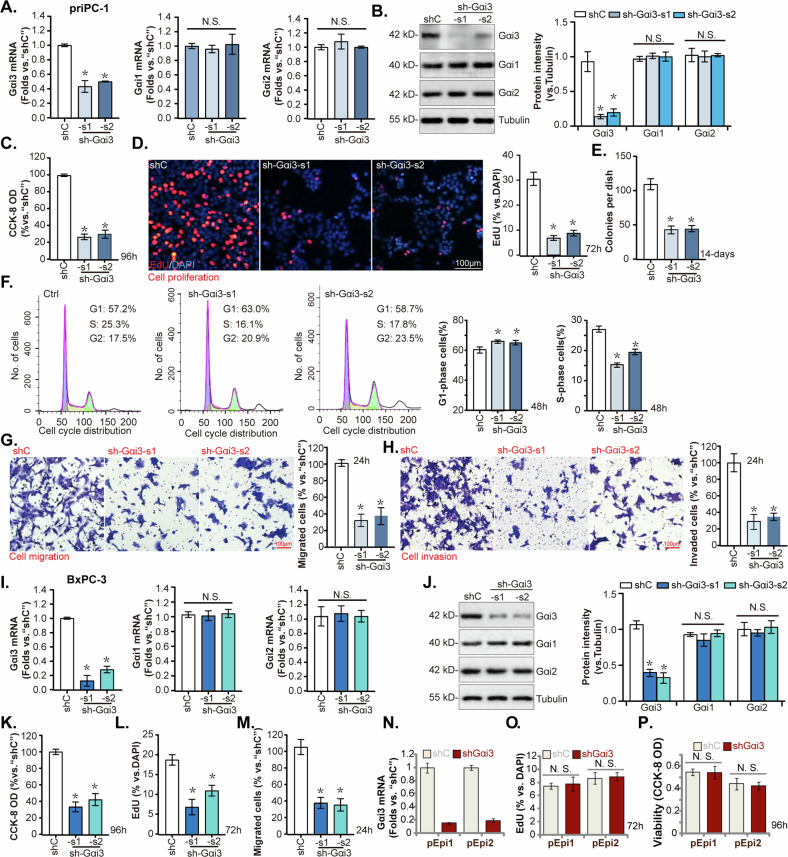
Gαi3 silencing by shRNA inhibits pancreatic cancer cell growth, cell cycle progression and mobility. The primary priPC-1 cells, the immortalized BxPC-3 cells and primary human pancreatic epithelial cells (pEpi1 and pEpi3) underwent stable transfection with lentivirus carrying Gαi3-targeting shRNAs (“sh-Gαi3”, with s1/s2 representing two distinct sequences) or a scramble control shRNA (“shC”), listed genes and proteins were tested (**A**, **B**, **I**, **J**, **N**). Cells were further cultivated for applied periods, cell viability (**C**–**K**, and **P**), proliferation (**D**, **L**, **O**), colony formation (**E**), cell cycle progression (**F**), migration (**G** and **M**), and invasion (**H**) were assessed. *P < 0.05 versus “shC” group. “N.S.” stands for *P* > 0.05. The experiments depicted in this figure were replicated five times (*n* = 5, biological repeats), consistently yielding similar results. Scale bar = 100 μm.

Given the notable upregulation of Gαi3 in human pancreatic cancer, its functional role in pancreatic cancer cells was further investigated. Primary human pancreatic cancer cells, designated “priPC-1,” as reported previously [29, 30], were subjected to infection with two lentiviral vectors encoding Gαi3-targeted short hairpin RNAs (shRNAs), specifically “sh-Gαi3-s1” and “sh-Gαi3-s2,” (with non-overlapping sequences [20, 24, 25, 28]), alongside a scramble nonspecific control shRNA (“shC”). Stable cells were established following selection in a puromycin-containing medium. Both *Gαi3* mRNA and protein levels were markedly reduced in the sh-Gαi3-bearing stable priPC-1 cells when compared with the control (“shC”), whereas the levels of Gαi1 and Gαi2 remained unchanged (Fig. 3A, B). A decrease in cell viability was observed in Gαi3-silenced priPC-1 cells, as measured by the CCK-8 assay (Fig. 3C). Additionally, Gαi3 knockdown by the targeted shRNAs significantly inhibited priPC-1 cell proliferation, as demonstrated by the reduction in EdU-positive nuclei (Fig. 3D), and cell colony formation ability (Fig. 3E). Cell cycle analysis revealed that Gαi3 shRNA induced G1-S phase arrest, indicated by increased proportion of cells in the G1 phase and concomitant decrease in the S phase (Fig. 3F). The impact of Gαi3 silencing on cell motility was assessed through “Transwell” (Fig. 3G) and “Matrigel Transwell” assays (Fig. 3H), revealing a substantial inhibition of priPC-1 cell motility.

The effects of Gαi3 silencing were also evaluated in the immortalized BxPC-3 pancreatic cancer cells, which were transduced with Gαi3 shRNA-expressing lentiviral particles. Following selection, stable cells exhibited depleted *Gαi3* mRNA and protein expression without affecting Gαi1 and Gαi2 levels (Fig. 3J, K). Similar to the functional results in priPC-1 cells, Gαi3 shRNA led to reduced cell viability (Fig. 3L), diminished proliferation (as evidenced by reduced EdU incorporation, Fig. 3M), and impaired migration (Fig. 3N) in BxPC-3 cells. In the primary human pancreatic epithelial cells, pEpi1 and pEpi2 [29], treatment with sh-Gαi3-s1 (“shGαi3”) also led to significant silencing of *Gαi3* mRNA (Fig. 3O). However, unlike in the cancer cells, this silencing of Gαi3 failed to impede proliferation (as measured by nuclear EdU incorporation, Fig. 3P) or affect viability (CCK-8 OD, Fig. 3Q) in these non-cancerous epithelial cells. These findings demonstrate that t shRNA-mediated Gαi3 silencing effectively inhibited pancreatic cancer cell growth, cell cycle progression and mobility in vitro.

### Gαi3 KO induces robust anti-pancreatic cancer cell activity in vitro

**Fig. 4:**
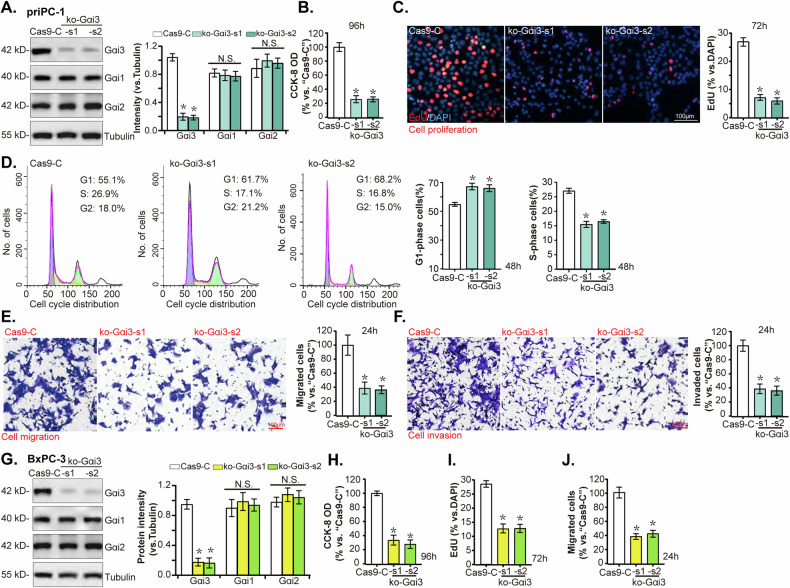
Gαi3 KO causes robust anti-pancreatic cancer cell activity. Primary priPC-1 cells and immortalized BxPC-3 cells were subjected to transduction with two distinct lentiviral CRISPR/Cas9 constructs targeting *Gαi3* for KO (“ko-Gαi3 “, with s1/s2 representing two distinct sgRNAs), in comparison to a control group transduced with an empty CRISPR/Cas9 vector (“Cas9-C”). Subsequently, stable cells were generated, and the listed proteins were evaluated (**A**–**G**). Cells were then cultured for additional specified time periods, cell viability (**B**–**H**), proliferation (**C**–**I**), cell cycle progression (**D**), migration (**E**–**J**), and invasion (**F**) were tested, with results quantified. *P < 0.05 versus “Cas9-C” group. “N. S.” stands for *P* > 0.05. The experiments depicted in this figure were replicated five times (*n* = 5, biological repeats), consistently yielding similar results. Scale bar = 100 μm.

To further support the role of Gαi3 in pancreatic cancer cells, the CRISPR/Cas9 genome editing technology was employed. The priPC-1 primary pancreatic cancer cells were individually transduced with two lentiviral CRISPR/Cas9-Gαi3-KO constructs (“ko-Gαi3-s1” and “ko-Gαi3-s2”), along with a CRISPR/Cas9 empty vector (“Cas9-C”). The two constructs contains sgRNA against different sequences against *Gαi3*. The introduction of ko-Gαi3 constructs led to a significant depletion of Gαi3 protein levels compared to the control group, without affecting the expression of Gαi1 and Gαi2 (Fig. 4A). Gαi3 KO resulted in decreased cell viability (Fig. 4B) and a reduction in cell proliferation, as indicated by a lower ratio of EdU-positive nuclei (Fig. 4C). Additionally, cell cycle analysis via PI-FACS revealed an increase in the proportion of G1-phase cells and a decrease in S-phase cells in the Gαi3 KO group, indicative of a G1-S phase arrest (Fig. 4D). Furthermore, Gαi3 KO markedly reduced the migratory (Fig. 4E) and invasive capabilities (Fig. 4F) of priPC-1 cells in vitro.

Similarly, in BxPC-3 pancreatic cancer cells, the application of ko-Gαi3-s1/s2 constructs resulted in pronounced downregulation of Gαi3 protein expression (Fig. 4G). Functional assays demonstrated that Gαi3 KO substantially suppressed cell viability (as evidenced by CCK-8 assay results, Fig. 4H), proliferation (indicated by reduced EdU-positive nuclei ratio, Fig. 4I), and migration (assessed by Transwell assays, Fig. 4J) in BxPC-3 cells. These findings unequivocally demonstrate that Gαi3 KO elicits strong anti-pancreatic cancer cell activity in vitro.

### Ectopic Gαi3 overexpression induces pro-cancerous activity in pancreatic cancer cells

**Fig. 5:**
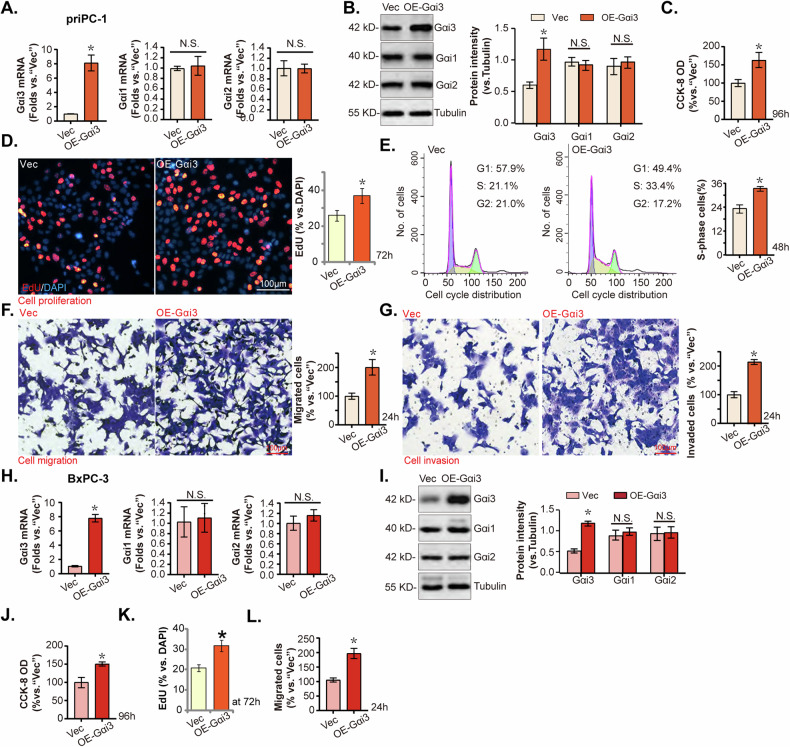
Ectopic Gαi3 overexpression induces pro-cancerous activity in pancreatic cancer cells. The primary priPC-1 cells and the immortalized BxPC-3 cells, expressing a lentiviral construct encoding Gαi3 (“OE-Gαi3”) or the empty vector (“Vec”), were formed, and the expression of listed genes and proteins were tested (**A**, **B**, **H**, **I**); Cells underwent further cultivation for indicated time periods, cell viability (**C**–**J**), proliferation (**D**–**K**), cell cycle progression (**E**), migration (**F**–**L**), and invasion (**G**) were tested, with results quantified. *P < 0.05 versus “Vec” group. “N.S.” stands for *P* > 0.05. The experiments depicted in this figure were replicated five times (*n* = 5, biological repeats), consistently yielding similar results. Scale bar = 100 μm.

Considering the observed anticancer effects following Gαi3 knockdown or KO in various pancreatic cancer cells. Ectopic overexpression of Gαi3 might exert opposite effects, potentially enhancing pancreatic cancer cell progression in vitro. To test this, priPC-1 cells were transduced with a lentiviral construct encoding Gαi3, as previously described [20, 22, 24, 25, 28]. Following puromycin selection, stable cells were established, designated as “OE-Gαi3,” exhibited significantly elevated levels of Gαi3 expression compared to control cells transduced with an empty vector (“Vec”) (Fig. 5A, B), while the expression levels of Gαi1 and Gαi2 remained consistent (Fig. 5A, B).

The overexpression of Gαi3 markedly increased cell viability, as determined by the CCK-8 assay (Fig. 5C), and significantly enhanced cell proliferation, tested by nuclear EdU incorporation (Fig. 5D). Additionally, cell cycle analysis indicated an increase in the percentage of cells in the S phase, suggesting accelerated cell cycle progression in OE-Gαi3 priPC-1 cells (Fig. 5E). Moreover, the migratory (Fig. 5F) and invasive (Fig. 5G) capabilities of priPC-1 cells were notably enhanced following Gαi3 overexpression. Similarly, BxPC-3 cells transduced with the “OE-Gαi3” lentiviral construct and subsequently selected with puromycin demonstrated a substantial upregulation of Gαi3 expression (Fig. 5H, I), without altering the expression of Gαi1 and Gαi2 (Fig. 5H, I). Gαi3 overexpression in BxPC-3 cells led to increased cell viability (Fig. 5J), augmented cell proliferation (Fig. 5K), and accelerated in vitro cell migration (Fig. 5L). These findings collectively underscore the pro-cancerous activity of Gαi3 in pancreatic cancer cells.

### Gαi3 is crucial for the activation of Akt-mTOR in pancreatic cancer cells

**Fig. 6:**
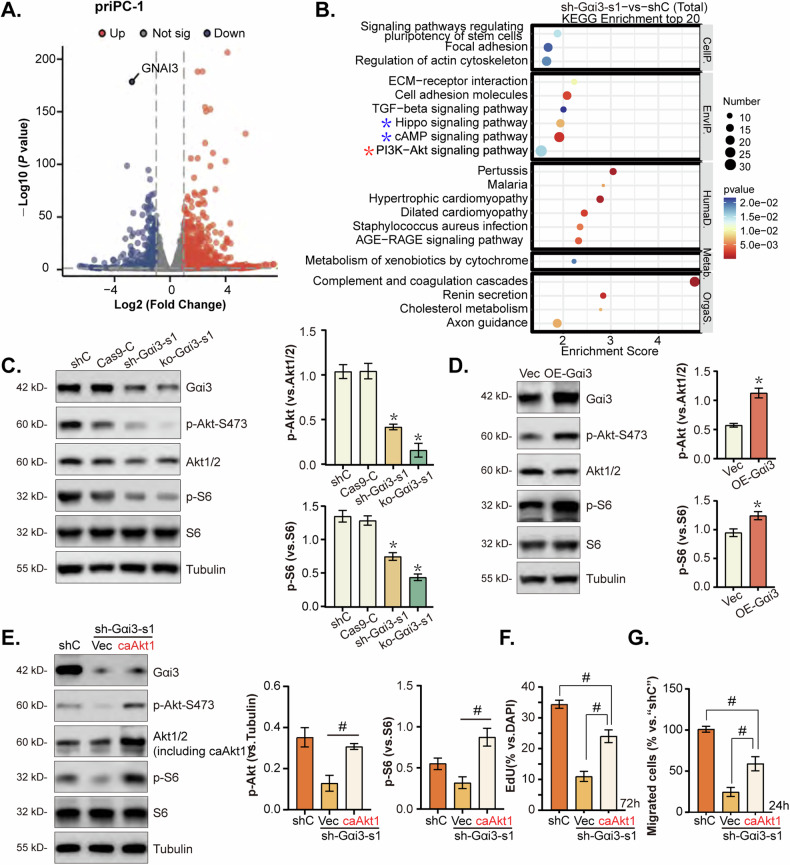
Gαi3 is crucial for the activation of Akt-mTOR in pancreatic cancer cells. RNA-sequencing (RNA-seq) was employed to analyze the gene expression profile of priPC-1 cells following the knockdown of Gαi3 using shRNA (“sh-Gαi3-s1”) in contrast to cells treated with a non-specific scramble shRNA (“shC”). This comparison elucidated a set of differentially expressed genes (DEGs), as depicted in volcano plots (**A**). Kyoto Encyclopedia of Genes and Genomes (KEGG) pathway analysis of the DEGs in sh-Gαi3-s1-expressing priPC-1 cells is shown (**B**). Western blot analysis was performed to examine the activation states of AKT-mTORC1 signaling in priPC-1 cells bearing the described genetic modifications (**C**, **D**). priPC-1 cells with“sh-Gαi3-s1” were engineered to express either a constitutively active Akt1 mutant (caAkt1, S473D) or a control vector (“Vec”), expression of the described proteins was assessed in these cells (**E**); Cells were then cultured for 24-72 h, cell proliferation (**F**) and migration (**G**) were measured by the indicated experiments, with results quantified. *P < 0.05 versus “shC”/“Cas9-C”/“Vec” group. ^#^*P* < 0.05. The experiments depicted in this figure were replicated five times (*n* = 5, biological repeats), consistently yielding similar results.

Our previous studies have established Gαi3’s association with receptor tyrosine kinases (RTKs) [15, 17–19, 23, 24] and non-RTK receptors [21–23], highlighting its role in facilitating the activation of the downstream Akt-mTOR signaling pathway. To further elucidate Gαi3’s involvement in Akt-mTOR pathway activation within pancreatic cancer cells, RNA-sequencing (RNA-seq) was employed to identify differentially expressed genes (DEGs) in Gαi3-silenced priPC-1 cells (Fig. 6A). Subsequent KEGG pathway analysis of these Gαi3-associated DEGs in Gαi3-knockdown priPC-1 cells revealed enrichment in various signaling pathways, notably the PI3K-Akt signaling pathway, as indicated by a red star (Fig. 6B). This enrichment supports that Gαi3 might play a crucial role in the Akt-mTOR signaling cascade in pancreatic cancer cells. Experimental data confirmed that knockdown or KO of Gαi3 significantly reduced the phosphorylation levels of Akt (Ser-473) and S6 (Fig. 6C). Conversely, ectopic overexpression of Gαi3 led to an increase in the phosphorylation of Akt (Ser-473) and S6 in priPC-1 cells (Fig. 6D), highlighting Gαi3’s critical role in Akt-mTOR pathway activation in the pancreatic cancer cells.

To determine if Akt-mTOR cascade activation is responsible for Gαi3-driven pancreatic cancer cell growth, priPC-1 cells expressing sh-Gαi3-s1 were transduced with the lentivirus encoding a constitutively active mutant of Akt1 (caAkt1). Introduction of caAkt1 restored Akt-S6 phosphorylation levels without affecting Gαi3 protein expression in these cells (Fig. 6E). Furthermore, quantified results showed that caAkt1 significantly counteracted the proliferation and migration inhibitions induced by Gαi3 knockdown in priPC-1 cells (Fig. 6F, G). These results collectively reinforce the notion that Gαi3’s promotion of pancreatic cancer cell growth is, in part, mediated through the activation of the Akt-mTOR signaling cascade.

### Gαi3 regulates PKA-Hippo-YAP signaling axis in pancreatic cancer cells

**Fig. 7:**
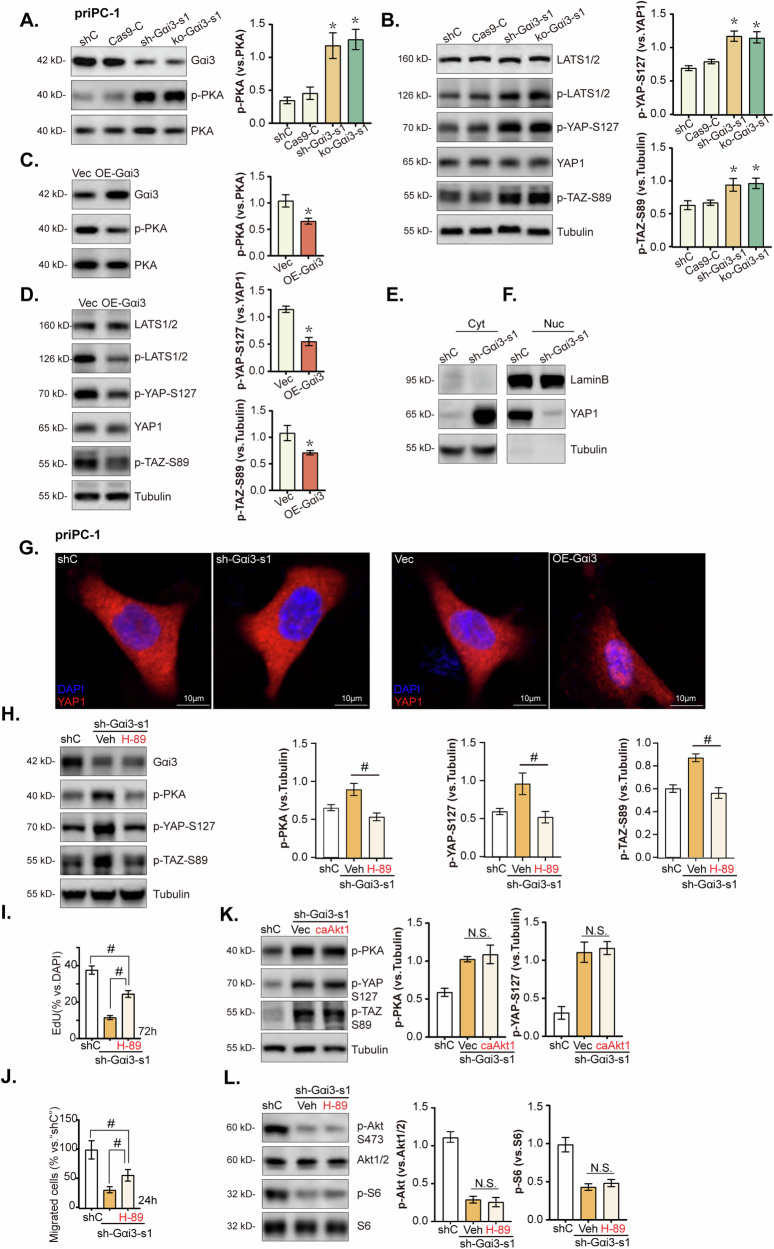
Gαi3 regulates PKA-Hippo-YAP signaling axis in pancreatic cancer cells. The primary priPC-1 cells stably expressing the Gαi3 shRNA (“sh-Gαi3-s1”), the CRISPR/Cas9-Gαi3-KO construct (“ko-Gαi3-s1”), the Gαi3-expressing lentiviral construct (“OE-Gαi3”), alongside their respective controls (“shC”, “Cas9-C” or “Vec”) were established, and the expression of listed proteins were tested (**A**–**D**). Primary priPC-1 cells stably expressing the Gαi3 shRNA (“sh-Gαi3-s1”), as well as the corresponding control (“shC”) underwent cytoplasmic/nuclear protein extraction, and listed proteins in the described cell fraction lysates were measured (**E**, **F**), cells were also subjected to immunofluorescence (IF) staining, with representative images presented (**G**). The priPC-1 cells expressingsh-Gαi3-s1 were treated with H-89 (a PKA inhibitor, 7 μM) or 0.1% DMSO as vehicle control (“Veh”), and expression of listed proteins was shown (**H**–**L**). After specified cultivation periods, cell proliferation and migration were examined, with results quantified (**I**, **J**). The priPC-1 cells with“sh-Gαi3-s1” were engineered to express either a constitutively active Akt1 mutant (caAkt1, S473D) or a control vector (“Vec”), expression of the described proteins was assessed by Western blotting (**K**). The designations “Cyt” and “Nuc” refer to cytoplasmic and nuclear proteins, respectively. *P < 0.05 versus “shC”/“Cas9-C”/“Vec” group. ^#^*P* < 0.05. Scale bars are provided as specified.

RNA-seq analysis combined with KEGG pathway exploration verified that Gαi3 is involved in modulating the cAMP and Hippo signaling pathways in pancreatic cancer cells, as highlighted by “blue stars” in Fig. 6B. Conventionally, Gαi3 is activated by GPCRs, leading to adenylate cyclase inhibition, cAMP levels reduction, and subsequent suppression of PKA (cAMP-dependent protein kinase) activity [36]. Its known that PKA activation can lead to the phosphorylation LATS (large tumor suppressor), a key kinase within the Hippo pathway [37–39]. This process results in the phosphorylation and subsequent inactivation of YAP (Yes-associated protein) and TAZ (transcriptional co-activator with PDZ-binding motif), the crucial downstream effectors of the Hippo signaling pathway, promoting their cytoplasmic retention and degradation, and inhibiting their oncogenic activity [40]. Our experiments demonstrated that the knockdown or KO of Gαi3 significantly increased PKA phosphorylation in priPC-1 cells (Fig. 7A). This is accompanied by elevated levels of phosphorylated LATS1/2, YAP (Ser-127), and TAZ (Ser-89), while total YAP1 levels remained unchanged (Fig. 7B). In contrast, overexpression of Gαi3 reduced PKA phosphorylation (Fig. 7C), leading to decreased phosphorylation of LATS1/2, YAP (Ser-127), and TAZ (Ser-89) in priPC-1 cells, without altering total YAP1 expression (Fig. 7D).

Given the known mechanism whereby Ser-127 phosphorylation triggers YAP 14-3-3 binding and its sequestration to the cytoplasm [41], we investigated the subcellular localization of YAP in Gαi3-deficient priPC-1 cells. Western blot assays revealed that Gαi3 knockdown markedly facilitated YAP’s nuclear-to-cytoplasmic translocation (Fig. 7E, F). IF assay corroborated these findings, showing that Gαi3 silencing promoted YAP cytoplasmic translocation, while its overexpression favored nuclear translocation (Fig. 7G). Hence, the suppression of PKA activity by Gαi3 inactivated the LATS1/2 kinase cascade within the Hippo pathway, thereby leading to YAP dephosphorylation and promoting its nuclear translocation in pancreatic cancer cells, indicating that Gαi3 mitigates PKA-induced inhibition of YAP/TAZ activity in pancreatic cancer cells.

To further elucidate the role of the PKA-Hippo-YAP axis in Gαi3’s oncogenic function in pancreatic cancer cells, we employed the specific PKA inhibitor H-89. Treatment with H-89 diminished the phosphorylation levels of PKA, LATS1/2, YAP (Ser-127), and TAZ (Ser-89) without affecting Gαi3 protein levels in sh-Gαi3-s1 priPC-1 cells (Fig. 7H). In priPC-1 cells, quantified results showed that H-89 alleviated the anti-proliferative (Fig. 7I) and anti-migratory effects (Fig. 7J) induced by Gαi3 silencing. Collectively, these results underscore the critical involvement of the PKA-Hippo-YAP axis in mediating Gαi3’s pro-cancerous activities in pancreatic cancer cells. Importantly, reintroducing Akt activation through caAkt1 had no impact on the PKA-Hippo-YAP pathway in Gαi3-silenced priPC-1 cells (Fig. 7K). Similarly, using the PKA inhibitor H-89 failed to restore the reduced Akt-S6 phosphorylation in sh-Gαi3-s1-expressing priPC-1 cells (Fig. 7L). These results demonstrate that the two pathways, each downstream of Gαi3, function independently in pancreatic cancer cells.

### TCF7L2 is a key transcription factor of Gαi3 in pancreatic cancer cells

**Fig. 8:**
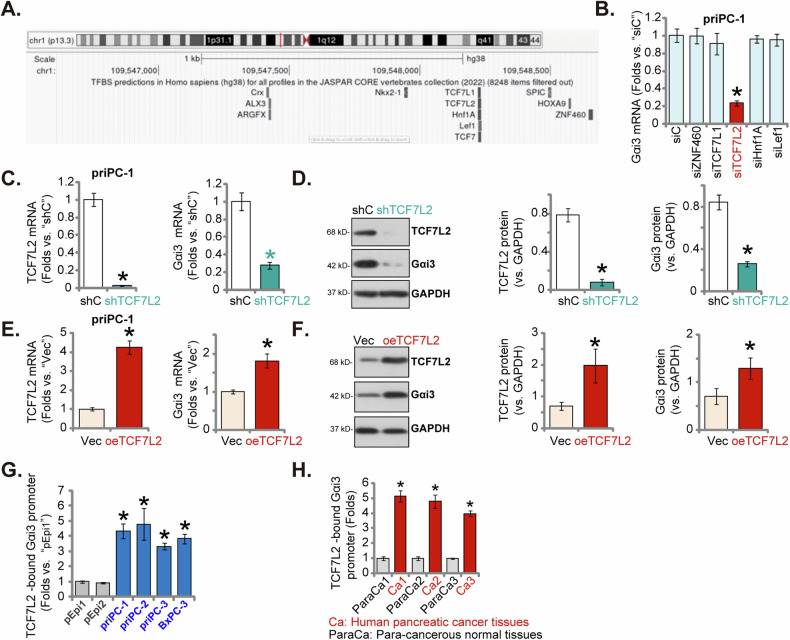
TCF7L2 is a key transcription factor of Gαi3 in pancreatic cancer cells. The JASPAR database was employed to predict the putative transcription factors of *Gαi3* (**A**). priPC-1 cells underwent transfection with siRNAs targeting various transcription factors, alongside a negative control siRNA (“siC”) for 48 h, *Gαi3* mRNA level was subsequently tested (**B**). priPC-1 cells were modified to express either a lentiviral shRNA targeting TCF7L2 (“shTCF7L2”), a scramble control shRNA (“shC”), a lentiviral construct overexpressing TCF7L2 (“oeTCF7L2”), or an empty vector (“Vec”), and the expression of listed mRNAs and proteins was examined (**C**–**F**). Chromatin immunoprecipitation (ChIP) assays presented the relative levels of TCF7L2-bound *Gαi3* promoter in the listed pancreatic cancer cells and primary human pancreatic epithelial cells (“pEpi”) (**G**) as well as in the designated pancreatic cancer tumor tissues (“T”) and matched adjacent normal pancreatic tissues (“N”) (**H**), with results quantified. The data were presented as mean ± standard deviation (SD). *P < 0.05 versus “siC”/“shC”/“Vec” /“pEpi”/“ParaCa” tissues. The experiments depicted in this figure were replicated five times (*n* = 5, biological repeats), consistently yielding similar results.

Observations of elevated *Gαi3* mRNA and protein levels in both pancreatic cancer tissues and cells prompted the investigation into a transcriptional regulatory mechanism underlying Gαi3 upregulation in pancreatic cancer. An initial search in the JASPAR transcription factor database [42] identified five transcription factors with the highest predicted binding affinity to the Gαi3 promoter: ZNF460, TCF7L2, TCF7L1, Hnf1A, and Lef1 (Fig. 8A). Subsequent experiments involved the design and transfection of specific siRNAs targeting these transcription factors into primary pancreatic cancer cells (priPC-1), with the aim to assess their impact on *Gαi3* mRNA levels. Notably, only the siRNA targeting TCF7L2 achieved significant silencing of *Gαi3* in priPC-1 cells, whereas siRNAs against the other transcription factors did not affect *Gαi3* expression (Fig. 8B). Further experimentation involved infecting priPC-1 cells with a lentivirus expressing TCF7L2 shRNA (“shTCF7L2”) or scramble control shRNA (“shC”) [43], followed by selection with puromycin to establish stable cells. *TCF7L2* mRNA (Fig. 8C) and protein (Fig. 8D) levels were significantly reduced in shTCF7L2 cells compared to control cells, accompanied by a concomitant decrease in *Gαi3* mRNA (Fig. 8C) and protein (Fig. 8D) expression. These results supported the role of TCF7L2 as a key transcriptional regulator of Gαi3 in pancreatic cancer cells.

To reinforce this hypothesis, priPC-1 cells were also infected with a lentivirus carrying a TCF7L2-expressing construct, and following puromycin selection, stable cells (“oeTCF7L2”) exhibited significant upregulation of both TCF7L2 and Gαi3 at the mRNA (Fig. 8E) and protein (Fig. 8F) levels. Importantly, ChIP assay demonstrated a significant increase in the binding of TCF7L2 protein to the *Gαi3* promoter region, as predicted in the JASPAR database, across various pancreatic cancer cells (Fig. 8G). Additionally, analysis of pancreatic cancer tissues from four patients (Patient-1#/-2#/-3#/-4#) revealed a significantly higher binding of TCF7L2 to the proposed *Gαi3* promoter compared to adjacent normal pancreatic tissues (Fig. 8H), suggesting that enhanced TCF7L2-*Gαi3* promoter interaction might be a fundamental mechanism contributing to Gαi3 overexpression in pancreatic cancer.

### Gαi3 shRNA suppresses pancreatic cancer xenograft growth in nude mice

**Fig. 9:**
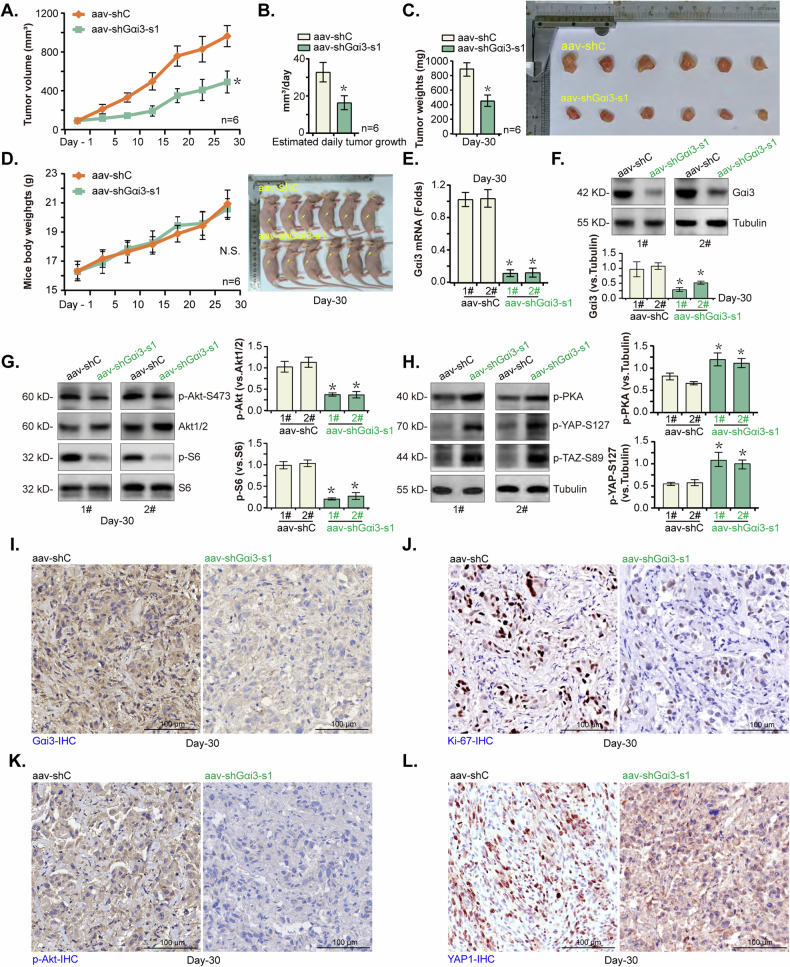
Gαi3 shRNA suppresses pancreatic cancer xenograft growth in nude mice. Mice bearing priPC-1 xenografts received intratumoral injections of adeno-associated virus (aav) expressing sh-Gαi3-s1 (“aav-shGαi3-s1”) or a scramble control shRNA (“aav-shC”), the volumes of the priPC-1 xenografts (**A**) and the mice body weights (**D**) were monitored and recorded at five-day intervals. The estimated daily priPC-1 xenograft growth was calculated (**B**). On the 30th day post-injection, all priPC-1 xenografts were excised and weighed (**C**). Tissue lysates from the harvested priPC-1 xenografts were obtained and expression of the listed mRNAs and proteins was tested (**E**–**H**). Furthermore, sections from these xenografts were processed for immunohistochemistry (IHC) staining to visualize the expression of Gαi3 (**I**), Ki-67 (**J**), p-Akt (Ser-473) (**K**), and YAP1 (**L**). The presented results are expressed as the mean ± standard deviation (SD), with the study including six mice per experimental group (*n* = 6). *P < 0.05 versus “aav-shC” group. “N. S.” stands for *P* > 0.05. Scale bar = 100 μm.

The potential role of Gαi3 on the growth of primary pancreatic cancer cells in vivo was investigated. Initially, priPC-1 cells were subcutaneously (s.c.) injected into the flanks of nude mice at a density of five million cells per mouse, following previously described methods [29, 30]. After three weeks, priPC-1 xenografts were successfully established with each xenograft approaching a volume of approximately 100 mm³ (“Day-0”). Subsequently, these priPC-1 xenograft-bearing nude mice received intratumoral injections of adeno-associated virus (aav) bearing shRNA against Gαi3 (“aav-shGαi3-s1” [25, 28]) or a scramble shRNA control (“aav-shC” [25, 28]). The injection was administered again after 48 h, with tumor volumes recorded every five days. The results demonstrated that aav-shGαi3-s1 injection significantly inhibited the growth of priPC-1 xenografts (Fig. 9A). The daily growth rate of the priPC-1 xenografts, calculated as described in previous studies [29, 30], showed again that aav-shGαi3-s1 injection potently impeded priPC-1 xenograft growth (Fig. 9B). On Day-30 of the experiment, all mice were euthanized, and the priPC-1 xenografts were excised and weighed, revealing that the xenografts from the aav-shGαi3-s1 group were significantly lighter and smaller than those ones from the aav-shC group (Fig. 9C). No significant difference in mice body weights was however observed between the two groups (Fig. 9D).

Further analysis was conducted on priPC-1 xenograft tissues from two mice (“1#/2#”) per group. Tumor tissue lysates were prepared, and subsequent assays showed a marked reduction in both mRNA and protein levels of Gαi3 in the aav-shGαi3-s1-treated xenograft tissues (Fig. 9E, F), accompanied by a significant decrease in phosphorylation of Akt (Ser-473) and S6 (Fig. 9G). In contrast, increased phosphorylation of PKA, YAP (Ser-127), and TAZ (Ser-89) were observed in the same set of tissues (Fig. 9H). Immunohistochemistry (IHC) staining further confirmed the silencing of Gαi3 protein in aav-shGαi3-s1 injected priPC-1 xenografts (Fig. 9I), along with a reduction in Ki-67-positive nuclei (Fig. 9J) and Akt activation (Fig. 9K). Furthermore, IHC images showed decreased nuclear localization of YAP1 in xenograft tissues following Gαi3 silencing (Fig. 9L). Collectively, these findings indicate that silencing of Gαi3 by aav-shGαi3-s1 injection effectively suppressed Akt-mTOR activation and YAP/TAZ activity in priPC-1 xenografts, and inhibiting tumor growth.

### Gαi3 KO impedes pancreatic cancer cell growth in vivo

**Fig. 10:**
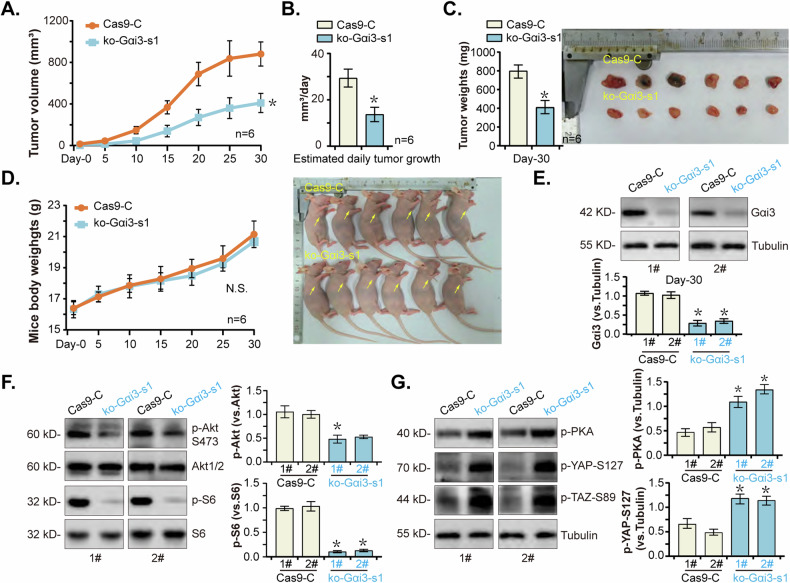
Gαi3 KO impedes pancreatic cancer cell growth in vivo. priPC-1 cells with the Gαi3 sgRNA1-expressing lenti-CRISPR/Cas9-KO construct (“ko-Gαi3-s1”) or the lenti-CRISPR/Cas9-KO construct (“Cas9-C”) were s.c. injected to flanks of the nude mice at a concentration of 20 million cells per mouse, with six mice included in each experimental group. The volumes of the priPC-1 xenografts (**A**) and the mice body weights (**D**) were monitored and recorded at five-day intervals. The daily growth rate of the priPC-1 xenografts was also assessed (**B**). Thirty days post-injection, the developed xenografts were surgically removed and tumor weights were recorded (**C**). Analysis of protein expression was conducted on tissue lysates derived from the harvested priPC-1 xenografts (**E**–**G**). The data were presented as mean ± standard deviation (SD, *n* = 6). *P < 0.05 versus “Cas9-C” group. “N. S.” stands for *P* > 0.05.

To further support the pivotal role of Gαi3 in pancreatic cancer cell growth in vivo, ko-Gαi3-s1 (sgRNA1) priPC-1 cells or “Cas9-C” cells, at 20 million cells per mouse, were *s.c*. injected into nude mice’s flanks. Comparative analysis demonstrated a significant inhibition in the growth of ko-Gαi3-s1 priPC-1 xenografts relative to the Cas9-C priPC-1 xenografts (Fig. 10A). The quantification of daily growth rates further validated the robust suppression of tumor growth in ko-Gαi3-s1 priPC-1 xenografts (Fig. 10B). Additionally, ko-Gαi3-s1 priPC1 xenografts were much lighter than Cas9-C priPC1 xenografts (Fig. 10C), whereas no significant difference was observed in the body weights (Fig. 10D). Gαi3 protein expression was depleted in ko-Gαi3-s1 priPC-1 xenografts (Fig. 10E), where the phosphorylation of Akt and S6 was remarkably decreased (Fig. 10F). Conversely, increased phosphorylation of PKA, YAP (Ser-127), and TAZ (Ser-89) were detected in ko-Gαi3-s1 priPC-1 xenografts (Fig. 10G). These results together reinforce the notion that Gαi3 KO significantly hindered the growth of priPC-1 xenografts in nude mice.

### Gαi3 expression is correlated with immune cell infiltration of pancreatic cancer

**Fig. 11:**
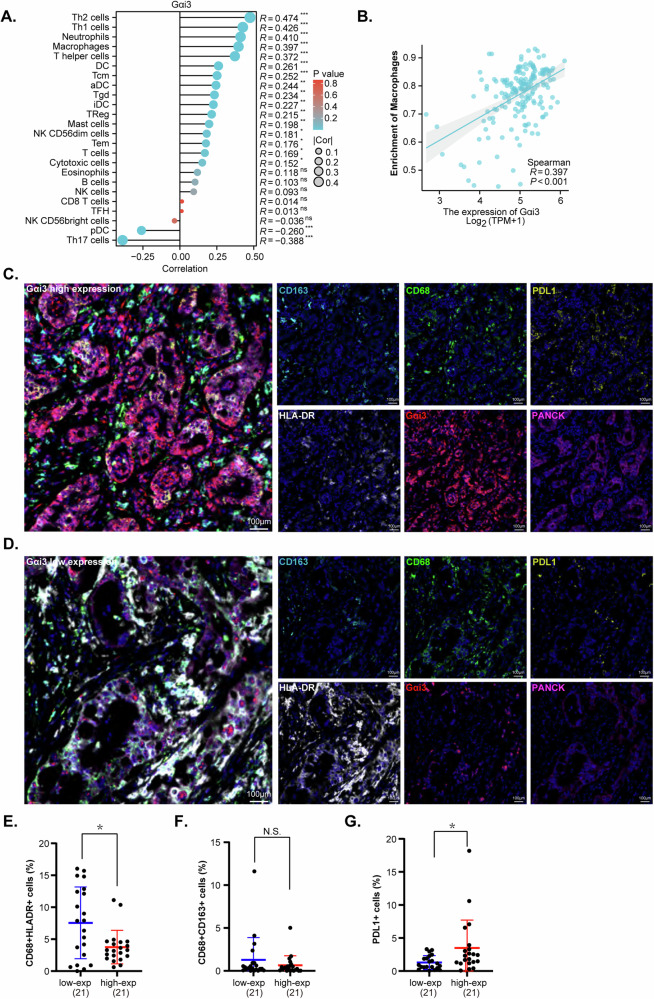
Gαi3 expression is correlated with immune cell infiltration of pancreatic cancer. Analysis based on TCGA-PAAD cohort shows the Gαi3’s association with various immune cell populations (**A**). The scatter plot shows the specific correlation between Gαi3 expression and the proportion of macrophages in pancreatic cancer tissues (**B**). Multiplex immunohistochemistry (mIHC) staining on tissue microarrays from pancreatic cancer patients, categorized by high (**C**) and low (**D**) Gαi3 expression levels, is presented alongside a color legend for the markers used. Different colors represent specific markers: Gαi3 in red, CD163 in turquoise, HLA-DR in white, CD68 in green, PANCK in pink, PDL1 in gold, and DAPI as the nuclear counter-stain. Quantitative analysis subsequent to the staining process allowed for the comparison of immune cell populations between the groups with high and low Gαi3 expression. The percentages of M1 macrophages (**E**), M2 macrophages (**F**), and PDL1 positive cells (**G**) were shown. The terms “low-exp” and “high-exp” are utilized to denote groups with low and high Gαi3 expression, respectively. *P < 0.05 versus “low-exp” group. “N. S.” stands for *P* > 0.05. Scale bar = 100 μm.

Upon delineating the critical role of Gαi3 in facilitating pancreatic cancer cell growth, a preliminary investigation was carried out to assess the impact of Gαi3 on the infiltration of immune cells within the pancreatic cancer tumor microenvironment (TME). The association between Gαi3 expression and immune cell presence was analyzed based on TCGA-PAAD cohort (Fig. 11A). We observed a significant correlation between Gαi3 expression and macrophage proportion in pancreatic cancer tissues, with a correlation coefficient (*R*) of 0.397 and a *P*-value of <0.001 (Fig. 11B). This finding prompted further investigation into the relationship between Gαi3 expression and the immune cell landscape within pancreatic cancer, employing multiplex immunohistochemistry (mIHC). mIHC staining facilitated simultaneous detection of six marker proteins: PANCK, Gαi3, HLA-DR, CD68, CD163, and PDL1, providing insights into the potential interaction between Gαi3 expression and immune cell infiltration (Fig. 11C, D).

Human pancreatic cancer tissues were categorized into two groups based on Gαi3 expression levels: “Gαi3-high expression” and “Gαi3-low expression”. mIHC analysis revealed a striking association between Gαi3 expression and the percentage of macrophages. Specifically, pancreatic cancer tissues characterized by higher Gαi3 expression showed an reduced proportion of macrophages, as evidenced by CD68 and HLA-DR positive staining, indicative of M1-type macrophage presence (Fig. 11E). This observation suggests that Gαi3 might influence the infiltration and activation of M1 macrophages within the pancreatic cancer TME. Whereas, the distribution of CD68 and CD163 positive staining cells, markers for M2-type macrophages, showed no significant difference between groups with low and high Gαi3 expression, hinting a limited effect of Gαi3 on the infiltration of M2 macrophages in the pancreatic cancer TME (Fig. 11F). Furthermore, an increase in PD-L1 positive cells was observed in pancreatic cancer tissues with higher Gαi3 expression (Fig. 11G). Considering the established role of PD-L1 as a critical immune checkpoint molecule capable of suppressing immune responses, its upregulation concurrent with increased Gαi3 expression may suggest a mechanism through which pancreatic cancer cells circumvent immune surveillance. These findings highlight the dual capacity of Gαi3 in modulating the macrophage phenotype within the pancreatic cancer microenvironment, and facilitating immune evasion, thereby highlighting its potential impact on the immune landscape of pancreatic cancer.

## Discussion

Pancreatic cancer stands as one of the deadliest malignancies, primarily due to delayed diagnosis, aggressive metastasis, and notable chemoresistance [44]. Despite advancements in screening methods and targeted therapies, the clinical outcomes for pancreatic cancer patients remain substantially unsatisfactory [44]. The heterogeneity of pancreatic cancer is marked by extensive genomic, proteomic, and epigenetic alterations affecting various core signaling pathways, such as RTKs, TGF-β receptor, Akt-mTOR, SWI/SNF complex, Wnt/Notch, JNK, and Hippo signaling [45–47]. These insights underscore the urgent need for innovative diagnostic and therapeutic targets for pancreatic cancer.

Our group has previously identified Gαi proteins, particularly Gαi1 and Gαi3, as crucial proteins in signal transduction for numerous RTKs [15–19] and non-RTK receptors [21–23]. Beyond facilitating oncogenic cascades triggered by activated cell surface receptors, Gαi1 and Gαi3 are overexpressed in various human malignancies, including glioma, osteosarcoma, cervical cancer, and nasopharyngeal carcinoma, where they correlate with poor prognosis [16, 24, 25, 28, 48]. Silencing or KO of Gαi3 has been shown to impede the progression of osteosarcoma, cervical cancer and glioma, while its overexpression promoted cancer cell growth [25, 28].

The current study also supported Gαi3’s role as a novel and promising therapeutic target of pancreatic cancer. Bioinformatics analyses reveal that Gαi3 expression is significantly overexpressed in human pancreatic cancer, correlating with poor prognosis, higher tumor grade, and advanced classification. Experimental validation confirmed the upregulated expression of Gαi3 in pancreatic cancer tissues and cell lines, contrasted with its lower expression in adjacent non-cancerous pancreatic tissues and pancreatic epithelial cells. Remarkably, depleting Gαi3 through shRNA or CRISPR-Cas9 gene editing led to significant anticancer effects in various pancreatic cancer cells and suppressed the growth of pancreatic cancer xenografts in vivo, while the ectopic overexpression of Gαi3 accelerated pancreatic cancer cell proliferation.

**Fig. 12:**
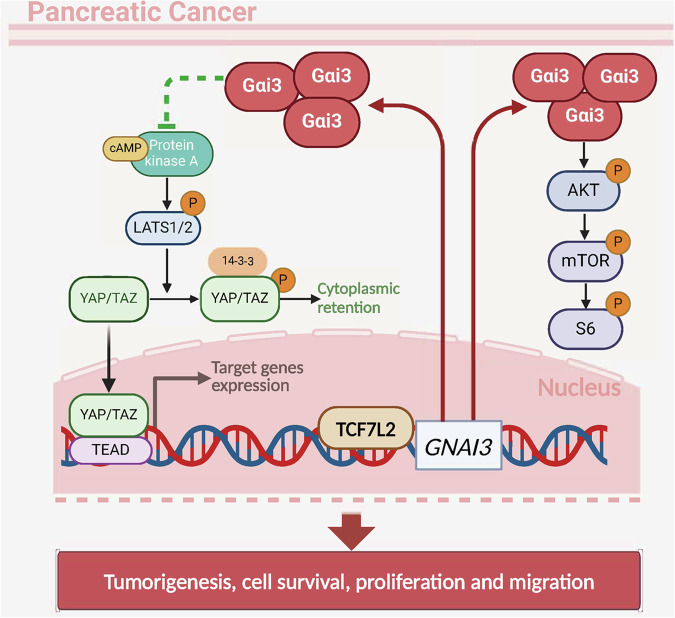
The proposed signaling carton of the present study. The current study emphasizes the complex role of Gαi3 in promoting the growth of pancreatic cancer cells by regulating the Akt-mTOR and PKA-Hippo-YAP signaling pathways. TCF7L2 is a crucial transcription factor for Gαi3, driving its upregulation in pancreatic cancer.

Previous research has established Gαi3’s interaction with multiple RTKs [15, 17–19, 23, 24] and non-RTK receptors [21–23], highlighting its contribution to the activation of the Akt-mTOR signaling cascade. The current study substantiates these findings by demonstrating that Gαi3-mediated pancreatic cancer cell growth is mediated, at least in part, by promoting the Akt-mTOR pathway activation. Comprehensive RNA-sequencing and subsequent KEGG pathway analyses conducted on Gαi3-silenced pancreatic cancer cells have highlighted its engagement in the PI3K-Akt signaling pathway. In primary human pancreatic cancer cells, Gαi3 shRNA or KO markedly diminished Akt-S6 phosphorylation, whereas ectopic Gαi3 overexpression exerted an opposite effect. In vivo studies revealed diminished Akt-S6 phosphorylation levels in Gαi3-depleted pancreatic cancer xenograft tissues. Introduction of caAkt1 to restore Akt-S6 activation compensated for the proliferation and migration inhibition of Gαi3-silencing pancreatic cancer cells, indicating a partial reliance of Gαi3-driven pancreatic cancer cell growth on Akt-mTOR pathway activation (Fig. 12).

Despite its role in mediating Akt-mTOR signaling, Gαi3 appears to be a critical regulator of the PKA-Hippo-YAP signaling axis as well. Canonically, Gαi protein activity, initiated by GPCR signaling, leads to adenylate cyclase (AC) inhibition, reducing cAMP levels and subsequently attenuating PKA activity [36, 49]. This process impacts the Hippo pathway, where PKA activation results in Hippo kinase LATS1/2 phosphorylation and consequent inactivation of the downstream effectors YAP/TAZ [40]. Activation of the Hippo pathway restricts cell growth and proliferation through the phosphorylation and subsequent inactivation of YAP/TAZ [50, 51]. Conversely, inactivation of this pathway can lead to dephosphorylation and then activation of YAP/TAZ, facilitating their nuclear translocation, where they bind to TEAD (transcriptional enhancer associate domain) family transcription factors, initiating transcriptional programs crucial for cell proliferation and migration [50, 51]. Notably, disruptions in Hippo signaling and the hyperactivation of YAP/TAZ are recognized as key factors in the development and chemoresistance of pancreatic cancer [47, 52].

RNA-seq analyses and bioinformatic studies on Gαi3-depleted pancreatic cancer cells have shed light on Gαi3’s potential role in modulating the PKA/Hippo signaling axis. Indeed, Gαi3 shRNA or KO led to enhanced activation of PKA and the Hippo pathway kinase LATS1/2, coupled with the inactivation of YAP/TAZ in primary pancreatic cancer cells. Conversely, ectopic overexpression of Gαi3 displayed an opposite effect. Moreover, increased activation of PKA and Hippo kinase LATS1/2, as well as the inactivation of YAP/TAZ, were significantly increased in Gαi3-depleted pancreatic cancer xenograft tissues. A critical observation from this study is that Gαi3 knockdown significantly promoted the nuclear-to-cytoplasmic translocation of YAP, whereas Gαi3 overexpression promoted its nuclear localization, without affecting YAP’s total expression level (Fig. 12). The inhibition of PKA activation through the use of H-89 reversed YAP/TAZ activation via dephosphorylation and ameliorated the proliferation and migration deficits observed in Gαi3-silencing pancreatic cancer cells. These findings underscore the significance of the PKA-Hippo-YAP signaling axis in facilitating the pro-tumorigenic activities of Gαi3 in pancreatic cancer. However, further research is required to elucidate the complex molecular interactions between the Akt-mTOR and PKA-Hippo-YAP pathways in the context of Gαi3-driven pancreatic cancer progression. Although our preliminary results supported the independence of the two pathways.

TCF7L2 has been implicated in the abnormal expression patterns and plays a pivotal role in the tumorigenesis, progression, and metastasis of various human malignancies. Wu et al. demonstrated that TCF7L2 transcriptionally induced the upregulation of SNHG11, thereby promoting proliferation and the epithelial-mesenchymal transition (EMT) in gastric cancer cells [53]. Jing et al. have shown that TCF7L2 is overexpressed in glioblastoma, correlating with diminished survival [54]. Moreover, Xiang et al. discovered that elevated expression of TCF7L2 in pancreatic cancer patients is frequently associated with poorer prognosis [55]. Our findings suggest that the transcriptional activation of Gαi3 by TCF7L2 may serve as a critical mechanism for Gαi3 overexpression in pancreatic cancer. In primary human pancreatic cancer cells, both mRNA and protein expressions of Gαi3 were significantly decreased following the silencing of TCF7L2, whereas ectopic overexpression of TCF7L2 led to an increase in Gαi3 expression. ChIP assay results underscored an enhanced interaction between the TCF7L2 protein and the *Gαi3* promoter region in both pancreatic cancer tissues and different cells, suggesting that this augmented binding may be a pivotal mechanism behind the upregulation of Gαi3 in pancreatic cancer (see Fig. 12).

Its known that the tumor microenvironment (TME), shaped by the intricate interactions between pancreatic epithelial/cancer cells and stromal cells, plays a significant role in metastasis and treatment resistance [56–58]. Our findings indicate a correlation between Gαi3 expression and the infiltration of immune cells within the pancreatic cancer TME. Through bioinformatic analysis and mIHC staining on pancreatic cancer tissue microarrays, we observed that elevated Gαi3 expression was associated with a reduced proportion of M1 macrophages, known for their anti-tumor activity [59], within the pancreatic cancer TME. However, the proportion of M2 macrophages, which presumably promote tumor growth and immune suppression, appeared unaffected [59, 60]. Additionally, an increase in PD-L1-positive cells was detected in pancreatic cancer tissues with higher Gαi3 expression, suggesting a mechanism for pancreatic cancer cell immune evasion by upregulating the surface expression of PD-L1, thereby inhibiting T cell function through interaction with PD-1 receptor on T cells [61–63].

Pancreatic cancer is characterized by a highly immunosuppressive tumor microenvironment (TME), which poses significant challenges for effective immunotherapy [64, 65]. While PD-1/PD-L1 inhibitors have demonstrated limited success in PDAC, the upregulation of PD-L1 remains a critical area of interest [66, 67]. PD-L1 expression can contribute to immune evasion by inhibiting the activity of cytotoxic T cells, and its upregulation may indicate an adaptive immune resistance mechanism within the tumor [67, 68]. Recent research has explored various combinatorial approaches to overcome the immunosuppressive TME in PDAC [69]. Combining PD-1/PD-L1 inhibitors with other modalities such as agents targeting other immune checkpoints has shown some promise in preclinical and early clinical studies [70–73]. Additionally, strategies aimed at modulating the TME, such as enhancing T cell infiltration, are being investigated to improve the response to immunotherapy [67, 72, 74]. Therefore, a comprehensive understanding of the complex interplay among Gαi3, PD-L1, and TME is important for developing effective immunotherapeutic strategies for this challenging disease.

Collectively, our study highlights Gαi3’s multifaceted role in promoting pancreatic cancer growth through the modulation of Akt-mTOR and PKA-Hippo-YAP signaling pathways, as well as its influence on the immune landscape within the pancreatic cancer TME. Given the pivotal role of Gαi3 in pancreatic cancer progression, targeting this protein or its downstream effectors represents a promising therapeutic avenue. Preclinical studies employing monoclonal antibodies or novel pharmacological agents could provide valuable insights into the feasibility and efficacy of such interventions. Furthermore, TCGA analysis revealed a significant association between Gαi3 overexpression and advanced disease characteristics, including patient age, histological grade, and nodal metastasis. Comprehensive studies examining the interactions of Gαi3 with key pathways, such as PI3K-Akt and Hippo-YAP, at various stages of disease progression will certainly help identify optimal time points for therapeutic intervention. These studies will advance our understanding of Gαi3’s role in pancreatic cancer and develop novel therapeutic strategies for patients with this devastating disease.

## Supplementary information

Figure S1. The uncropped blotting images.

## Data Availability

All data are available upon request.
