# Supplementary figures and images for: Identification of Gαi3 as a promising molecular oncotarget of pancreatic cancer
Source: Cell Death Dis. 2024 Sep 30;15(9):699. doi: 10.1038/s41419-024-07079-6 (PMC11442978; doi:10.1038/s41419-024-07079-6)

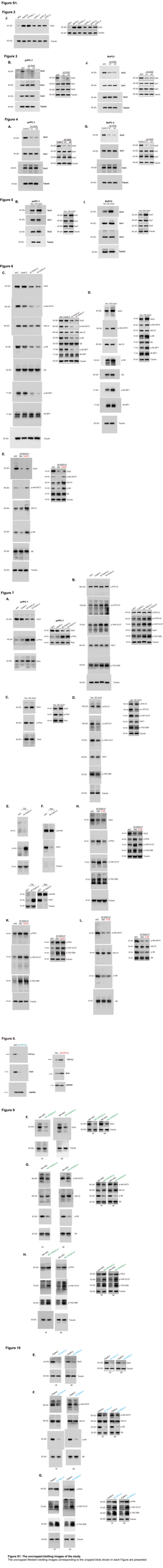

Supplement: Supplementary file 1 — Figure S1. The uncropped blotting images. [file 41419_2024_7079_MOESM1_ESM.pdf]
